# Host–pathogen coevolution promotes the evolution of general, broad-spectrum resistance and reduces foreign pathogen spillover risk

**DOI:** 10.1093/evlett/qrad051

**Published:** 2023-10-16

**Authors:** Samuel V Hulse, Janis Antonovics, Michael E Hood, Emily L Bruns

**Affiliations:** Department of Biology, University of Maryland at College Park, College Park, MD, United States; Department of Biology, University of Virginia, Charlottesville, VA, United States; Department of Biology, Amherst College, Amherst, MA, United States; Department of Biology, University of Maryland at College Park, College Park, MD, United States

**Keywords:** coevolution, host–pathogen, spillover, general resistance, specific resistance

## Abstract

Genetic variation for disease resistance within host populations can strongly impact the spread of endemic pathogens. In plants, recent work has shown that within-population variation in resistance can also affect the transmission of foreign spillover pathogens if that resistance is general. However, most hosts also possess specific resistance mechanisms that provide strong defenses against coevolved endemic pathogens. Here we use a modeling approach to ask how antagonistic coevolution between hosts and their endemic pathogen at the specific resistance locus can affect the frequency of general resistance, and therefore a host’s vulnerability to foreign pathogens. We develop a two-locus model with variable recombination that incorporates both general resistance (effective against all pathogens) and specific resistance (effective against endemic pathogens only). With coevolution, when pathogens can evolve to evade specific resistance, we find that the regions where general resistance can evolve are greatly expanded, decreasing the risk of foreign pathogen invasion. Furthermore, coevolution greatly expands the conditions that maintain polymorphisms at both resistance loci, thereby driving greater genetic diversity within host populations. This genetic diversity often leads to positive correlations between host resistance to foreign and endemic pathogens, similar to those observed in natural populations. However, if resistance loci become linked, the resistance correlations can shift to negative. If we include a third linkage-modifying locus in our model, we find that selection often favors complete linkage. Our model demonstrates how coevolutionary dynamics with an endemic pathogen can mold the resistance structure of host populations in ways that affect its susceptibility to foreign pathogen spillovers, and that the nature of these outcomes depends on resistance costs, as well as the degree of linkage between resistance genes.

## Introduction

Natural populations are frequently confronted with foreign pathogen spillovers, where a host becomes infected with a novel pathogen (Borremans et al., 2019). Warming climates and habitat destruction are driving changes in species distributions, which can increase spillover risks via increased pathogen exposure rates (Jones et al., 2008). Understanding the drivers of susceptibility to foreign pathogens is therefore essential. At the species level, the phylogenetic distance between naive and ancestral hosts is a key predictor of host shift potential, where pathogens are typically more infectious against closely related hosts (B. J. Parker et al., 2017; Streicker et al., 2010). However, within species, populations often harbor genetic variation in disease resistance (Baker & Antonovics, 2012; Laine, 2004; Thrall et al., 2001), including variation in resistance to diseases with which they share no coevolutionary history (Antonovics et al., 2002; Best & Kerr, 2000; Savage & Zamudio, 2011). Processes that drive the evolution of higher host resistance to endemic pathogens could therefore increase resistance to foreign pathogens if that resistance is positively transitive, that is, resistance to endemic pathogens is positively correlated with resistance to foreign pathogens.

Recently, Lerner et al. (2021) found a significant positive correlation between genetic resistance to an endemic fungal pathogen and to a related foreign pathogen in the herbaceous plant *Silene vulgaris*. Based on this data, the authors hypothesized that resistance to both foreign and endemic pathogens might be partially determined by the same locus. Given this framework, if the correlation between resistance to foreign and endemic pathogens is positive, selection for increased resistance to endemic pathogens could lead to decreased risk from spillover pathogens. The authors then developed a model that assumed resistance transitivity was determined by a single pleiotropic locus and found that the degree of resistance correlation (as measured by the slope of the regression between susceptibility to foreign and endemic pathogens when looking across replicates within a population) can affect the risk of foreign pathogen invasions. Importantly, they found that negative transitivity can significantly increase a population’s susceptibility to spillovers, even when the population’s average level of resistance to foreign pathogens is the same. Negative transitivity means a subset of the population will remain more susceptible to foreign pathogens than endemic pathogens, creating an ecological niche for foreign pathogens to invade and persist. However, we do not yet understand the evolutionary forces that could influence the slope of the resistance correlation or maintain genetic diversity for resistance to foreign pathogens. One potential clue lies in the presence of a few notable and repeatable outliers in Lerner’s data, where some host genotypes were more susceptible to the foreign pathogen than to the endemic pathogen. These outliers suggest some genotypes may have specific resistance against the endemic pathogen but lack more general forms of resistance that could protect against foreign pathogens.

Plants and animals have evolved general forms of resistance that are effective against a wide range of pathogens. Examples of general resistance mechanisms include inflammation in animals, as well as thickening of cell walls, and production of antimicrobial peptides in plants (Nürnberger et al., 2004). Since these mechanisms are broadly effective, general resistance should protect hosts against foreign pathogens. However, hosts have also evolved highly specific forms of resistance that are triggered by a single species of endemic pathogen (or often even a specific pathogen genotype) (Märkle et al., 2022). Previously, using an adaptive dynamics approach, we found that strong selection from a single, invariant endemic pathogen can favor the evolution of specific resistance and drive the loss of general resistance, leaving populations more vulnerable to foreign pathogens (Hulse et al., 2023). However, our prior model did not account for host–pathogen coevolution.

Specific resistance is particularly vulnerable to host–pathogen coevolution because it generates a strong positive selection for pathogen genotypes that can evade this resistance. In plants, this dynamic has been formalized with the gene-for-gene model (Flor, 1956). In the gene-for-gene model, a specific resistance gene in the host (referred to as an “*R*-gene”) recognizes a particular effector molecule secreted by a pathogen that is a gene product of an “Avirulence” gene (referred to as an “*Avr*” gene). Mutations in the *Avr* gene that avoid recognition by the *R* gene (referred to as the virulent form, or “*vir*”) allow the pathogen to successfully infect resistant hosts, often driving cyclical dynamics in host resistance and pathogen virulence (Tellier & Brown, 2007). Many natural systems have been shown to exhibit temporally and spatially variable selection for resistance due to gene-for-gene dynamics (Laine, 2005; Thrall & Burdon, 2003; Thrall et al., 2012). In agriculture, the introduction of new *R* genes is reliably followed by the rapid evolution of corresponding pathogen virulence (Carson, 2011; Miller et al., 2020). In contrast, general forms of resistance in crop species are often considered to be more “durable” over time and do not generate the same rapid coevolutionary response from pathogens (Mundt, 2014; Poland et al., 2009). However, few studies have investigated how coevolutionary dynamics at specific resistance loci affect the evolution of general resistance (Koskella & Parr, 2015; Koskella et al., 2011).

Here, we develop a two-locus host–pathogen model to investigate the joint evolution of two forms of innate resistance: general resistance (affecting both endemic and foreign pathogens) and specific resistance (affecting only endemic pathogens). We first ask how the introduction of a pathogen that can evolve in response to specific, but not general resistance shapes the evolution of both forms of resistance. For models to be relevant to natural systems, evolutionary outcomes must be described in terms measurable for empiricists. Therefore, we use our model to estimate the family-level correlation in resistance (transitivity) to endemic and foreign pathogens, a measure easily quantifiable in natural populations, where the exact loci underlying resistance are often unknown. Our results suggest that the degree of recombination between general and specific resistance strongly affects evolutionary outcomes. We therefore introduced a third locus that modifies the recombination rate to determine whether selection favors the evolution of linkage between general and specific resistance. We find that coevolution expands the range of conditions under which general resistance can evolve and can drive both positive and negative resistance correlations, depending on resistance costs and genetic linkage between resistance loci.

## Methods

We develop a compartmental model (Equations 1 and 2) that is loosely based on the dynamics of the anther-smut disease (caused by *Microbotryum silenes-inflatae*) on the herbaceous plant *Silene vulgaris*. In this system, transmission is frequency dependent, and infection causes sterilization without significant effects on mortality (Thrall et al., 1995). For simplicity, we assume haploid hosts, with a locus for general resistance and a locus for specific resistance. General resistance gives hosts a moderate level of resistance against all pathogen genotypes and species, while specific resistance gives hosts a high level of resistance against a single endemic pathogen genotype. Each locus has an allele (*g* and *s*) which confer no resistance benefits and no costs, as well as an allele (*G* and *S*) which confer both resistance benefits and fecundity costs, giving our model four possible host genotypes (Table 1).

**Table 1.** Resistance levels and birthrates for each genotype.

Host genotype	Host fecundity	Avirulent endemic transmission	Virulent endemic pathogen transmission	Foreign pathogen transmission
*GS*	$\left(1-c_{G})(1-c_{S}\right)$	$\beta (1-r_{G})(1-r_{S})$	$\beta (1-r_{G})$	$\beta (1-r_{G})(1-r_{f})$
*Gs*	$1-\;c_{G}$	$\beta (1-r_{G})$	$\beta (1-r_{G})(1-r_{v})$	$\beta (1-r_{G})(1-r_{f})$
*gS*	$1-\;c_{S}$	$\beta (1-r_{S})$	β	$\beta (1-r_{f})$
*gs*	1	β	$\beta (1-r_{v})$	$\beta (1-r_{f})$

In our model, the endemic pathogen has two genotypes: avirulent (*Avr*), which is sensitive to both forms of resistance, and virulent (*vir*) which is only sensitive to general resistance. Note that we follow the conventions of the gene-for-gene notation and use the term “virulent” to mean the specific ability to cause disease on a specifically resistant host (Flor, 1956; Thompson & Burdon, 1992), rather than as amount of damage caused to the host, as it is more commonly used in the animal literature.

### Resistance costs and benefits

The baseline host–pathogen transmission rate is given by β, which can then be reduced by host resistance or costs of virulence (Table 1). We denote the resistance conferred by general resistance by $r_{G}$ and the resistance conferred by specific resistance by $r_{S}$. We also include each host genotypes’ susceptibility to a foreign pathogen. Following spillovers, foreign pathogens are often poorly adapted to their hosts (Lee et al., 2017). The foreign pathogen’s transmission rate can thus be reduced from the baseline by $r_{f}$, for all host genotypes, which can be further reduced by general resistance.

Each form of resistance carries a cost to hosts in the form of a reduction in fecundity ($c_{G}$ and $c_{S}$). The resistance benefits of general and specific resistance, as well as their costs interact multiplicatively. The virulent genotype incurs a cost of virulence, given by $r_{v}$, which reduces transmission on hosts lacking specific resistance (we notate this cost as $r_{v}$ rather than *c*_*v*_ since it affects the pathogen’s transmission rate rather than host’s fecundity). This framework is based on Leonard’s “soft selection” gene-for-gene model (Leonard, 1994).

### SI dynamics

We define the transmission matrix B by $B_{ij}=\beta_{ij}$ where $\beta_{ij}$ is the transmission rate of the *j*th pathogen to the *i*th host (Table 1). Similarly, we define the resistance cost vector c by letting $c_{i}$ be the resistance costs of the *i*th host (Table 1). Incorporating these assumptions into a frequency-dependent SI model (Ross, 1916), we have the following system of ordinary differential equations.


$$\begin{array}{l} \begin{array}{l} \dot{x}=n_{s}\;(b\circ f)M-x\left((kn+\mu )1+\frac{1}{n}By\right)\; \end{array} \end{array}$$ (1)

$$\begin{array}{l} \begin{array}{l} \dot{y}=y\left(\frac{1}{n}B^{T}x-\mu 1\right)\; \end{array} \end{array}$$ (2)

Here, x is the vector of uninfected host genotype abundances, and y is the vector of infected host abundances (for each pathogen genotype). The host population size (infected and non-infected) is given by *n*, while $n_{s}$ is the total number of uninfected hosts. The vector 1 represents the vector where every element is 1. The parameters *k* and µ represent the coefficient of host density-dependent growth and death rate, respectively. For uninfected hosts, new births occur via random mating, given by $n_{s}\;(b\circ f)M$, where M is a two-locus mating matrix (we later expand this to three loci, see below) with recombination rate ρ (see below). An example of two-locus mating matrix is given in Supplementary Table S1. Finally, b is the vector of maternal birthrates for each parental pair, and f is the probability of each parental pair (elementwise vector multiplication is denoted by ∘).

### Mating matrix

Let *G* denote the genotype matrix, where $G_{ij}\in \left\{0,1\right\}$ denotes the *j*th allele of the *i*th genotype. We assume that the loci are sequentially ordered, giving a total of $2^{n}$ genotypes, where *n* is the number of loci. We must then calculate the probability of maternal genotype *i*, and paternal genotype *j* producing offspring genotype *k* for all $i,j,k\;\in \left\{1,\ldots ,2^{n}\right\}$. To do so, we calculate the probability that an offspring will receive their allele at each locus from a particular parent.

Let $P(M_{i})$ be the probability that an offspring receives their *i*th allele from their mother and $P(F_{i})$ be the probability that their *i*th allele is from their father. We assume that $P(M_{1})=P(F_{1})=0.5$. Next, let $\rho_{i},i\in \left\{1,\ldots ,n-1\right\}$ be the recombination rate between locus *i* and *i* + 1. We can then calculate the following allele origin probabilities inductively.


$$\begin{array}{l} P(M_{i})=1-\rho_{i}\;allele\;at\;position\;i-1\;is\;from\;the\;mother \end{array}$$

$$\begin{array}{l} P(M_{i})=\rho_{i}\;allele\;at\;position\;i-1\;is\;from\;the\;father \end{array}$$

For each locus, the paternal origin probabilities are then given by $P(F_{i})=1-P(M_{i})$. Since we assume recombination events are independent, the probability of the offspring’s genotypes coming from a particular sequence is the product of the probability of each allele. For any maternal, paternal, offspring combination, i, j, k, a subset of parental locus combinations will yield offspring genotype k. As these events are mutually exclusive, the probability of offspring k given maternal genotype i and paternal genotype j is the sum of the probabilities of each of these combinations. We then collapse the resulting $2^{n}\times 2^{n}\times 2^{n}$ tensor to a matrix with dimensions $2(2^{n})\times 2^{n}$, such that the first index represents the parental genotype combination and the second index the offspring genotype.

### Transitivity slope

**Figure 1.**
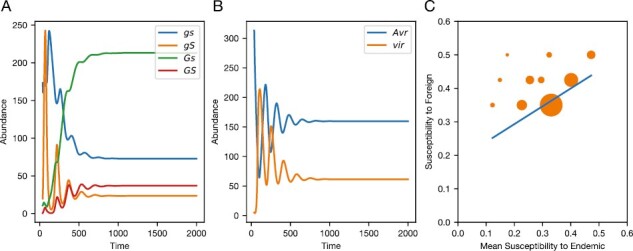
Example host–pathogen dynamics and resistance transitivity. (A) Abundances for each uninfected host genotype over time. (B) Abundance of infected hosts for both the avirulent (*Avr*) and virulent (*vir*) pathogen genotypes over time. (C) Resistance transitivity given the equilibrium host and pathogen frequencies. Here, each dot represents a full-sib family, and its position on each axis is determined by the average susceptibility of each family to the endemic (averaged over each pathogen genotype) and foreign pathogen. The size of each dot represents the likelihood of each full-sib family given the equilibrium frequencies of each host genotype. The blue line shows a linear regression of these points, weighted by their frequency. The transitivity slope is given by this regression. So that the transitivity slope represents relative infectivity from baseline, we set $r_{f}=0$. For all panels, the parameters used were $k=0.001,\mu =0.2,b=1,\rho =0.05,\beta =0.5,r_{G}=0.3,r_{S}=0.9,c_{g}=0.1,c_{s}=0.2$.

We used the transitivity slope as a measure of the family-level relationship between susceptibility to the foreign and endemic pathogens. With four host genotypes there are 16 full-sib families, given by the paternal–maternal genotype combinations. We first calculate the average susceptibility to the foreign and endemic pathogen for each full-sib family. For full-sib family *i*, the family-level susceptibility to pathogen *j* is given by


$$\begin{array}{l} S_{ij}=\;〈B_{*j}\;|\;M_{i*}〉\Rightarrow S=BM\; \end{array}$$

where $B_{*j}$ is the transmission rate of pathogen *j* across each host genotype, and $M_{i*}$ is the vector of offspring probabilities for parental pair *i*, given by the mating matrix. We consider the susceptibility to the endemic pathogen be the average across the *Avr* and *vir* genotypes, weighted by the relative abundance of each pathogen genotype. For susceptibility to foreign pathogens, we consider the case where foreign pathogens have the same baseline transmission as endemic pathogens $\left(r_{f}=0\right)$ but note that because the foreign pathogen is not included in the dynamics, the relative magnitude and sign of the transitivity slope is not affected. The transitivity slope is then computed by taking a weighted linear regression of all families’ average susceptibility to the endemic versus the foreign pathogen, weighted by likelihood of each family, f. For simulations which yielded only a single type of full-sib family, we considered the transitivity to be zero. An example model outcome, showing the convergence of host and pathogen frequencies, as well as the transitivity slope in given in Figure 1.

### Numerical analysis

Due to the high dimensionality and nonlinearity of our system, we were unable to find analytic expressions for our equilibria. We therefore used the modified Powell method for numerical root finding to estimate equilibrium points for our system, implemented with the Scipy 1.10.1 package in Python 3.11.2. (Powell, 1964; Virtanen et al., 2020). To ensure these estimated equilibria converged to interior (nondisease-free) equilibria, we first used the explicit Runge–Kutta method to compute numerical solutions, implemented with the SciPy 1.10.1. Each simulation was run to *t* = 5,000. We then used the final point of these simulations as an initial guess for numerical root finding. To test the stability of these equilibria, we computed finite difference approximations of the Jacobian matrix at equilibria. We also computed numerical solutions to *t* = 10,000 and checked whether the solution at the end points were consistent with our numerical root finding (see Supplementary Material).

### Parameter space investigated

Throughout our investigation, we assumed that general resistance was weaker than specific resistance $\left(r_{G}=0.3,\;r_{S}=0.9\right)$, as is often the case for plant pathogens (Poland et al., 2009). All other parameters were initially fixed as follows: $k=0.001,\mu =0.2,b=1,\rho =0.05,\beta =0.5,r_{f}\;=0.1$. We began all simulations with the same initial conditions: 100 uninfected individuals of each of the four host genotypes and 10 hosts infected with the avirulent endemic pathogen genotype. For simulations with coevolution, we began with an additional 1 host infected with the virulent endemic pathogen genotype. We ran three series of simulations. First, we only included the avirulent endemic pathogen genotype to establish baseline equilibria without coevolution. In these simulations, we varied the cost of general resistance, and the cost of specific resistance ($0\leq c_{G}\leq 0.2,\;0\leq c_{S}\leq 0.4$, values outside this range generally resulted in fixation at both loci). Next, we ran coevolutionary simulations including both endemic pathogen genotypes, also varying the costs of each form of resistance. Finally, we ran a set of coevolutionary simulations where we varied the cost of virulence instead of the cost of specific resistance ($0\leq c_{G}\leq 0.2,\;0\leq r_{v}\leq 0.3$, $c_{S}$ was fixed at 0.2). For this last set of simulations, we opted to vary the cost of general resistance rather than the cost of specific resistance because the cost of general resistance had a more pronounced effect on evolutionary outcomes (see results). If polymorphism was maintained at both resistance loci, we calculated the degree of linkage disequilibrium (using Lewontin’s $D^{\prime }$, see Supplementary Material), and the transitivity slope.

To assess the generality of our results, we ran a series of simulations with alternative assumptions. First, we investigated the impact of using density-dependent transmission, with a baseline transmission rate of β= 0.001 to account for the different units of density-dependent transmission. We then tested the effects of the hard selection gene-for-gene model (Leonard, 1977), where the costs of virulence for the virulent pathogen genotype are applied equally against all host genotypes. Next, we investigated the effects of stronger general resistance, setting the strength of resistance, $r_{G}$ to 0.5 (opposed to our baseline of 0.3). Finally, we tested the effects of the recombination rate, testing both full recombination ρ= 0.5 and complete linkage, ρ= 0.

### Evolution of linkage

Our results showed that the equilibria conditions were impacted by the degree of recombination, with the special case of complete linkage (ρ= 0) leading to markedly different outcomes than ρ= 0.05 (see Results). We therefore tested whether selection would favor the evolution of linked general and specific resistance. To do this, we added a third unlinked locus which modified the degree of linkage between *G* and *S*. We assume that this locus is unlinked from the general and specific resistance loci $\left(\rho_{1}=0.5\right)$. Each genotype’s recombination rate between general and specific resistance $\left(\rho_{2}\right)$ is then determined by the maternal linkage modifier allele. This framework allows us to model the ability for an allele which modifies recombination to invade. We then repeated the previous coevolutionary simulations with the additional linkage modifier locus, testing whether an allele which results in complete linkage $\left(\rho_{2}=\;0\right)$ between general and specific loci would evolve in a background of intermediate recombination $\left(\rho_{2}=\;0.05\right)$.

Our numerical approximation successfully converged for parameters examined. For nearly all parameters, the equilibria were asymptotically stable, per our numerical analysis. Exceptions to this generally occurred when costs of specific resistance or virulence were near zero and were likely due to numerical stability issues (see Supplementary Figures S1 and S2). The results found though numerical root finding were qualitatively identical to our results found with numerical integration.

## Results

### The effect of coevolution on host evolution

**Figure 2.**
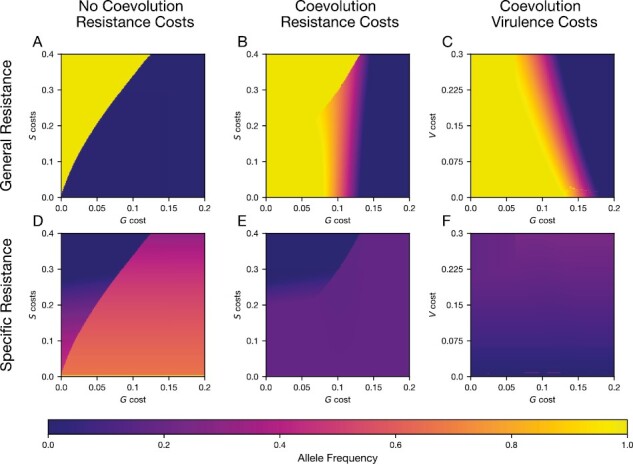
Host allele frequencies at equilibrium as a function of costs. Colors on the top row (A–C) show the equilibrium frequency of the general resistance (*G*) allele, with warmer colors representing higher frequency. Colors on the bottom row (D–F) show the equilibrium frequency of the specific resistance (*S*) allele. (A, D) Frequency of the *G* and *S* alleles in simulations without coevolution, where the cost of general resistance, $c_{G}$ and the cost of specific resistance, $c_{S}$ are varied. (B, E) Frequency of the *G* and *S* alleles in simulations with coevolution, where the cost of general resistance, $c_{G}$ and the cost of specific resistance, $c_{S}$ are varied. (C, F) Frequency of the *G* and *S* alleles in simulations with coevolution where the cost of general resistance, $c_{G}$ and the cost of virulence, $r_{v}$ are varied. Other parameters: $k=0.001,\mu =0.2,b=1,\rho =0.05,\beta =0.5,r_{G}=0.3,r_{S}=0.9$.

Without coevolution, general and specific resistance were highly exclusionary, where hosts typically maintained only one form of resistance (contrast Figure 2A and D). Maintaining resistance at both loci was only possible when costs for both general and specific resistance were low (Figure 2A and D, bottom left corner). Allowing coevolution through the introduction of the virulent pathogen genotype expanded the range of conditions where general resistance was maintained (compare Figure 2A and B) and reduced the frequency of specific resistance (compare Figure 2D and E). This expansion of general resistance in response to coevolution was a robust result that occurred with density-dependent transmission (Supplementary Figure S4A–C), alternative cost structuring for pathogen virulence (Supplementary Figure S4D–F), as well as with both moderate $\left(r_{G}=0.3\right)$ and high ($r_{G}=0.5$; Supplementary Figure S4G–I) levels of general resistance.

Coevolution also expanded the range of conditions that led to stable polymorphism for general resistance. In our baseline scenario, when general resistance was moderate $\left(r_{G}=0.3\right)$, polymorphism at the *G* locus could only be maintained with coevolution (Figure 2D, orange region). Higher levels of general resistance could drive the maintenance of *G*/*g* polymorphism in the absence of coevolution, but coevolution greatly expanded the region of stable polymorphisms (Supplementary Figure S4G–H). Coevolution led to the maintenance of simultaneous polymorphisms for general resistance (Figure 2B, orange region) and specific resistance loci (Figure 2E), enabling the maintenance of all four host alleles.

The frequencies of host resistance alleles depended on the costs of both general and specific resistance. Intermediate general resistance costs enabled polymorphism at both the general and specific resistance loci (Figure 2B, orange zone). However, costs of specific resistance did not have as strong an effect on the evolutionary outcomes. Very high costs of *S*$\left(c_{S}>0.25\right)$ could lead to the loss of specific resistance altogether (Figure 2E, dark blue zone) unless general resistance was also costly.

The cost of pathogen virulence influenced the equilibrium frequencies of both general and specific resistance (Figure 2C and F). Lower virulence costs increased the frequency of general resistance and decreased the frequency of specific resistance. With low virulence costs, the virulent genotype reached higher frequencies, and consequently selection for specific resistance was reduced since it was ineffective against the virulent genotype. As a result, the relative benefit of general resistance increased. Conversely, higher costs of virulence decreased the frequency of the virulent pathogen genotype, increased the frequency of *S*, and decreased the frequency of *G*. Applying virulence costs to all host types did not strongly affect host allele frequencies (Supplementary Figure S4D–F).

### The effects of recombination on general resistance

**Figure 3.**
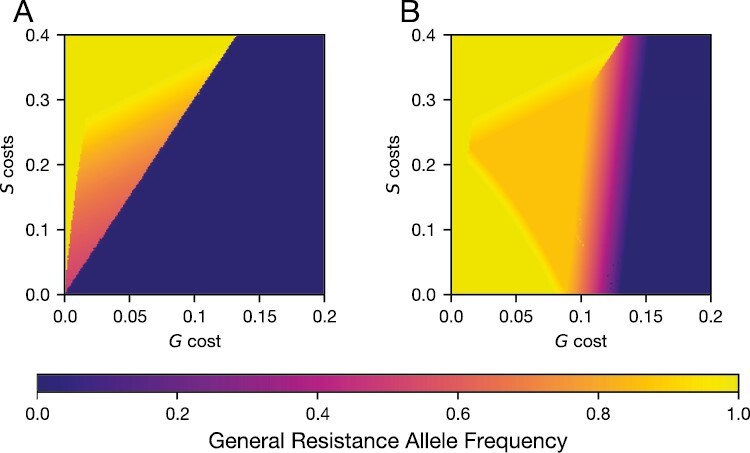
Frequency of the general resistance allele in simulations with no recombination. (A) Frequency of general resistance without coevolution. (B): Frequency of general resistance with coevolution. These panels are equivalent to Figure 1A and B. All other parameters besides the recombination rate (ρ= 0) are the same as in Figure 2.

Increasing the recombination rate above our baseline ρ= 0.05 did not cause any substantial changes to allele frequencies (Supplementary Figure S4J–L). The extreme case of complete linkage (ρ=0, Figure 3), generated different outcomes, maintaining polymorphism in *G* even in the absence of coevolution (Figure 3A). In this case, the *Gs* and *gS* genotypes were maintained under frequency-dependent selection, while the *GS* and *gs* genotypes were lost. As a result, both general and specific resistance were polymorphic, even though only two genotypes were sustained. Coevolution expanded the region of polymorphism (Figure 3B, orange area), but as in the no coevolution scenario, only two genotypes (*Gs* and *gS*) were maintained.

### Determinants of resistance correlation

The cost of general resistance has a significant effect on the transitivity slope. If general resistance was lost or fixed, all genotypes had the same level of susceptibility to a foreign pathogen. Therefore, only parameters generating *G*/*g* polymorphism led to non-zero transitivity slopes. Since without coevolution, the *G* allele was either fixed or lost, coevolution was a requirement for non-zero transitivity slopes (assuming recombination). However, with intermediate *G* frequencies, there was a considerable region of positive transitivity (Supplementary Figure 4).

Transitivity was highest when the frequency of *G* was at near 50%, and the cost of specific resistance was low (Supplementary Figure 4C; see also Supplementary Figure 2B and C for reference to the frequency of *G*). We found that the parameters most important to determining the slope of resistance transitivity were those that influenced the frequency of *G*, principally the cost of general resistance, $c_{g}$ (Supplementary Figure 4C). With low general resistance costs, *G* became fixed, and the transitivity slope was zero (as there is no variation in susceptibility to foreign pathogens). Generally, we found the highest transitivity values when the cost of specific resistance was low; however, this may be less informative, as there was little between-family variation in susceptibility to the endemic pathogen.

**Figure 4.**
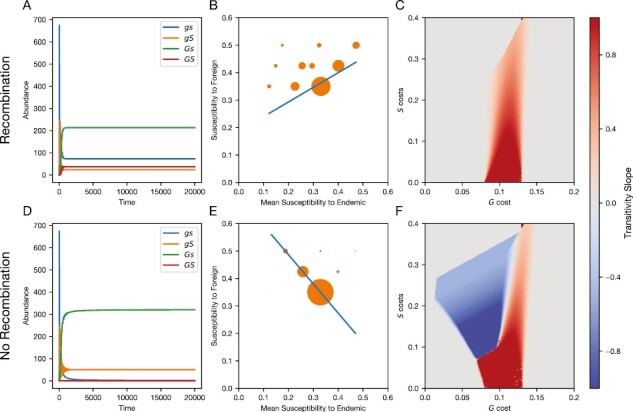
Resistance transitivity and the effects of the recombination rate. (A–C) Results with the default recombination rate (ρ= 0.05), while panels (D–F) assume no recombination (ρ= 0) (A, D) Host genotype abundances for (A) recombining and (D) nonrecombining hosts. (B, E) Resistance transitivity across full-sib families for (C) recombining and (F) nonrecombining hosts. Each dot represents the average susceptibility values for each full-sib family. The size of each symbol is proportional to the probability of randomly sampling each family. For A, B, D and E, $c_{G}=0.1$ and $c_{S}=0.2$. (E, F) Transitivity slope for full-sib families with coevolution for different costs of general resistance, $c_{G}$ and costs of specific resistance, $c_{S}$. All parameters other than the recombination rate are the same as Figure 2.

A notable exception to the pattern of positive transitivity occurred when general and specific resistance were in complete linkage (ρ=0, Supplementary Figure 4D–F). With recombination rates above ~0.02, and coevolution, *Gs* and *gs* were the most common genotypes (Figure 4A). However, without recombination, this switched to *Gs* and *gS* (Figure 4D), which were more fit without the loss of gametes to the costly double-resistant *GS* and highly susceptible *gs* genotypes. Given the high susceptibility to the foreign pathogen and low susceptibility to the endemic of *gS*, along with the even resistance of *Gs*, this resulted in negative transitivity (Figure 4E). The loss of recombination also led to a sharp boundary between positive and negative transitivity, where small changes in the cost of general resistance could significantly increase a population’s susceptibility to spillover pathogens. Without recombination, positive transitivity is generated when *gs* and *Gs* become the most common genotypes (as opposed to *Gs* and *gS* for negative transitivity), driving an overall positively correlated trend.

### Evolution with a linage modifier allele

**Figure 5.**
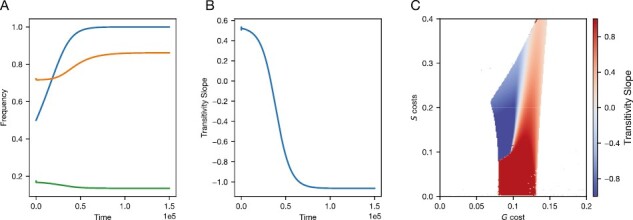
Allowing the evolution of recombination can result in complete linkage between general and specific resistance. Here, one linkage modifier allele causes a recombination rate ( ρ = 0.05), while the other results in no recombination ( ρ = 0). (A) Allele frequencies for general resistance *G* (orange line), and specific resistance *S* (green line), following the introduction of a linkage modifier allele (blue line). (B) Transitivity slope over time. (C) Parameters where the linkage modifier evolves to fixation, with colors showing the transitivity slope (as in Figure 4). White denotes regions where selection on the linkage modifier was neutral, owing to the lack of *G* polymorphism. All panels: parameters: $k=0.001,\mu =0.2,b=1,\rho =0.05,\beta =0.5,r_{G}=0.3,r_{S}=0.9$. Panels (A) and (B): $c_{g}=0.1,\;c_{s}=0.2$.

We found that a linkage modifier that eliminated recombination between general and specific resistance could invade and sweep to fixation in all cases where general resistance was polymorphic (Figure 5). In regions without general resistance polymorphism (also corresponding to regions with positive linage disequilibrium, see Supplementary Figure S3), we found neutral selection on the two recombination alleles (white region, Figure 5C, Supplementary Figure S2). However, if selected for, the time required for the linkage modifier allele to go from introduction (50%) to fixation was significantly longer than it took for general and specific resistance to approach equilibrium (t ∼ 5,000 vs. t ∼ 100,000, Figure 4A vs. Figure 5A) showing that while selection for tight linkage is often positive, it is also relatively weak. In many of these cases, the loss of recombination led a population that would otherwise have positive transitivity to switch to negative transitivity (Figure 5B).

## Discussion

Emerging infectious diseases pose an increasing threat to agriculture and human health (El-Sayed & Kamel, 2020; Jones et al., 2008). While spillovers typically occur between phylogenetically related host species (I. M. Parker et al., 2015; Longdon et al., 2014), species often exhibit standing genetic variation for resistance to foreign pathogens (Gilbert et al., 2018; Marmor et al., 2006; McCarthy et al., 2011). This observation begs the question: what has led to the maintenance of this foreign pathogen resistance? Is it an evolutionary response to constant spillover events, or can it be explained as the outcome of coevolutionary dynamics with an endemic pathogen? Our results show that host–pathogen coevolution, in the absence of a foreign pathogen, can drive the evolution of a broad-spectrum, general resistance and the maintenance of genetic diversity within host populations.

In a previous study (Hulse et al., 2023), we found that under most conditions, evolution with an invariant endemic pathogen favored specific resistance while suppressing general resistance, maintaining a population’s susceptibility to foreign pathogens. However, our current results show that if an endemic pathogen can coevolve in response to specific resistance, general resistance is maintained more frequently, decreasing spillover risk. In both cases of foreign pathogen invasions and coevolution with endemic pathogens, the relative utility of specific resistance relative to general resistance is reduced, leading to more situations where general resistance can be maintained. With coevolution, hosts maintained general resistance even when specific resistance had no costs, unlike in models without coevolution (Hulse et al., 2023). This is highly relevant for plant systems, where specific resistance costs have often been hard to detect (Brown, 2002; Korves & Bergelson, 2004). While our model was based on a frequency-dependent disease of wild plants, our key findings were robust to variation in transmission mode, as well as varying levels of general resistance and different forms of pathogen virulence costs.

We also found that coevolution can lead to stable polymorphism for general resistance. In noncoevolving populations, moderate to low resistance is either fixed or lost, and polymorphism can only be maintained with high levels of resistance via ecological feedbacks (Antonovics & Thrall, 1994). Yet, we found that across all levels of general resistance, the addition of a coevolving pathogen expanded the region of polymorphism and led to greater genotypic diversity. This occurred even though coevolution occurred at the specific resistance locus, not the general resistance locus. Our results could therefore help explain how populations maintain genetic variation for foreign pathogens for which they do not share a coevolutionary history (Laine, 2004; Thrall et al., 2001).

Resistance transitivity, the correlation between resistance to endemic and foreign pathogens, can be readily quantified in natural populations where the loci driving resistance are unknown. Moreover, Lerner et al. (2021) demonstrated that the transitivity slope can strongly influence a foreign pathogen’s ability to invade and persist in a population. Negative transitivity can facilitate spillovers by generating an ecological niche where foreign pathogens can outcompete endemic pathogens. We found that non-zero resistance transitivity only occurred under conditions that allowed general resistance polymorphism. Furthermore, with even modest recombination, we only observed positive transitivity. This result recapitulates the results found by Lerner et al. (2021) for *Silene vulgaris*. However, with complete linkage, resistance transitivity could become strongly negative because hosts either carried only the specific resistance gene or the general resistance gene, but rarely both. When the recombination rate is allowed to evolve, selection is neutral or favors complete linkage. This result is synergistic with empirical evidence, as in plants, *R* genes are often clustered together in the genome (Hulbert et al., 2001; Meyers et al., 2005).

In our model, general resistance was conferred by a single gene that conveyed moderate levels of broad-spectrum resistance. While general resistance is more typically considered to be a polygenic trait, with many loci of small effect (Poland et al., 2009), there is considerable value in understanding the evolutionary dynamics at a single general resistance locus, before adding additional complexity. Indeed, our results disrupt a common view of general resistance as evolutionarily “durable,” and unaffected by pathogen coevolution (Brown, 2015). Moreover, major-gene general resistance has been described in plants, for example, the *PigmR* gene in rice is a typical nucleotide-binding, leucine-rich repeat (NLR) gene that protects against a large breadth of *Magnaporthe orzye* genotypes (Deng et al., 2017). However, future expansion of the model to explicitly consider a quantitative form of general resistance could provide new insights, particularly regarding the evolution of linkage.

Our coevolutionary model for specific resistance was based on gene-for-gene interactions, which are common in plant-pathogen systems (Thompson & Burdon, 1992). It is possible that a different infection genetics, such as matching alleles (Grosberg & Hart, 2000), could alter the outcome. In a matching allele model, the ability to infect one host genotype comes at the cost of the complete inability to infect other host genotypes. Polymorphism in both hosts and pathogens can be maintained without costs due to strong negative frequency-dependent fluctuating selection (Agrawal & Lively, 2002); therefore, it would be unlikely for matching alleles to favor costly general resistance. In contrast, gene-for-gene interactions require both costs of resistance and virulence for the maintenance of polymorphism (Tellier & Brown, 2007). In the future, expansion of the model to multilocus gene-for-gene dynamics (Sasaki, 2000) or intermediate infection genetic models (Boots et al., 2014) could provide additional insights.

A key prediction from our model is that gene-for-gene coevolution with specific resistance can drive the evolution of general resistance. The best way to test our predictions would be through either experimental evolution or time-shift experiments in natural populations, as of both these approaches lead to direct estimates of dynamics. In a time-shift experiment with naturally occurring bacteria and phage inside leaves, Koskella and Parr (2015) found that while the phage rapidly adapted over time to track their local bacteria (suggestive of specific infectivity), the bacterial hosts evolved a more general form of resistance over time. Similarly, Frickel et al. (2016) found that the experimental coevolution of algae and viruses led to the evolution of general resistance in the algae. These results would seem to support our model, showing that general resistance is favored in the face of rapid coevolution. However, more recently, Lewis et al. (2022) found the opposite trend, where *Caenorhabditis elegans* coevolved with a bacterial pathogen were more susceptible to foreign strains than the control hosts. In all cases however, the underlying infection genetic system is not known, making it challenging to draw direct comparisons with theory.

Our results demonstrate how pairwise coevolution between hosts and pathogens at a single resistance locus can ripple out and affect evolutionary dynamics at other resistance loci via feedbacks between host and pathogen, with broader consequences for cross-species transmission. Previous research has demonstrated the importance of feedback between general and specific resistance (Frank, 2000; Hulse et al., 2023), although less is known about how these dynamics are affected by coevolution. Coevolutionary arms-race dynamics have been shown in many systems (Grenfell et al., 1995), such as the gene-for-gene system in plants (Flor, 1956), with major implications for natural populations as well as agricultural systems. However, the focus has mostly been on the evolution of specific resistance genes themselves. By incorporating the evolution of general resistance into a coevolutionary model, our results demonstrate how coevolving endemic pathogens can make host populations more resilient to spillover.

## Supplementary Material

qrad051_suppl_Supplementary_Tables_S1_Figures_S1-S2

## Data Availability

All code used to generate our data is available via GitHub at https://github.com/svhulse/gfg-model.

## References

[CIT0001] Agrawal, A., & Lively, C. M. (2002). Infection genetics: Gene-for-gene versus matching-alleles models and all points in between. Evolutionary Ecology Research, 4(1), 91–107.

[CIT0002] Antonovics, J., Hood, M., & Partain, J. (2002). The ecology and genetics of a host shift: Microbotryum as a model system. The American Naturalist, 160(Suppl 4), S40–S53. 10.1086/342143 18707452

[CIT0003] Antonovics, J., & Thrall, P. (1994). The cost of resistance and the maintenance of genetic polymorphism in host–pathogen systems. Proceedings of the Royal Society of London, Series B: Biological Sciences, 257(1349), 105–110. 10.1098/rspb.1994.0101

[CIT0004] Baker, C., & Antonovics, J. (2012). Evolutionary determinants of genetic variation in susceptibility to infectious diseases in humans. PLoS One, 7(1), e29089. 10.1371/journal.pone.0029089 22242158 PMC3252296

[CIT0005] Best, S. M., & Kerr, P. J. (2000). Coevolution of host and virus: The pathogenesis of virulent and attenuated strains of Myxoma virus in resistant and susceptible European rabbits. Virology, 267(1), 36–48. 10.1006/viro.1999.0104 10648181

[CIT0006] Boots, M., White, A., Best, A., & Bowers, R. (2014). How specificity and epidemiology drive the coevolution of static trait diversity in hosts and parasites. Evolution, 68(6), 1594–1606. 10.1111/evo.12393 24593303 PMC4257575

[CIT0007] Borremans, B., Faust, C., Manlove, K. R., Sokolow, S. H., & Lloyd-Smith, J. O. (2019). Cross-species pathogen spillover across ecosystem boundaries: Mechanisms and theory. Philosophical Transactions of the Royal Society of London, Series B: Biological Sciences, 374(1782), 20180344. 10.1098/rstb.2018.0344 31401953 PMC6711298

[CIT0008] Brown, J. K. M. (2002). Yield penalties of disease resistance in crops. Current Opinion in Plant Biology, 5(4), 339–344. 10.1016/S1369-5266(02)00270-4 12179968

[CIT0009] Brown, J. K. M. (2015). Durable resistance of crops to disease: A Darwinian perspective. Annual Review of Phytopathology, 53(1), 513–539. 10.1146/annurev-phyto-102313-045914 26077539

[CIT0010] Carson, M. L. (2011). Virulence in oat crown rust (*Puccinia coronata* f Sp Avenae) in the United States from 2006 through 2009. Plant Disease, 95(12), 1528–1534. 10.1094/PDIS-09-10-0639 30732001

[CIT0011] Deng, Y., Zhai, K., Xie, Z., Yang, D., Zhu, X., Liu, J., Wang, X., Qin, P., Yang, Y., Zhang, G., Li, Q., Zhang, J., Wu, S., Milazzo, J., Mao, B., Wang, E., Xie, H., Tharreau, D., & He, Z. (2017). Epigenetic regulation of antagonistic receptors confers rice blast resistance with yield balance. Science, 355(6328), 962–965. 10.1126/science.aai8898 28154240

[CIT0012] El-Sayed, A., & Kamel, M. (2020). Climatic changes and their role in emergence and re-emergence of diseases. Environmental Science and Pollution Research International, 27(18), 22336–22352. 10.1007/s11356-020-08896-w 32347486 PMC7187803

[CIT0013] Flor, H. H. (1956). The Complementary Genic Systems in Flax and Flax Rust**Joint Contribution from the Field Crops Research Branch, Agricultural Research Service, United States Department of Agriculture and the North Dakota Agricultural Experiment Station. In M. Demerec (Ed.), Advances in genetics (Vol. 8, pp. 29–54). Academic Press. 10.1016/S0065-2660(08)60498-8

[CIT0014] Frank, S. A. (2000). Specific and non-specific defense against parasitic attack. Journal of Theoretical Biology, 202(4), 283–304. 10.1006/jtbi.1999.1054 10666361

[CIT0015] Frickel, J., Sieber, M., & Becks, L. (2016). Eco-evolutionary dynamics in a coevolving host–virus system. Ecology Letters, 19(4), 450–459. 10.1111/ele.12580 26898162

[CIT0016] Gilbert, B., Bettgenhaeuser, J., Upadhyaya, N., Soliveres, M., Singh, D., Park, R. F., Moscou, M. J., & Ayliffe, M. (2018). Components of *Brachypodium distachyon* resistance to nonadapted wheat stripe rust pathogens are simply inherited. PLoS Genetics, 14(9), e1007636. 10.1371/journal.pgen.1007636 30265668 PMC6161853

[CIT0017] Grenfell, B. T., Dobson, A. P., & Moffatt, H. K. (1995). Ecology of infectious diseases in natural populations. Cambridge University Press.

[CIT0018] Grosberg, R. K., & Hart, M. W. (2000). Mate selection and the evolution of highly polymorphic self/nonself recognition genes. Science, 289(5487), 2111–2114. 10.1126/science.289.5487.2111 11000110

[CIT0019] Hulbert, S. H., Webb, C. A., Smith, S. M., & Sun, Q. (2001). Resistance gene complexes: Evolution and utilization. Annual Review of Phytopathology, 39(1), 285–312. 10.1146/annurev.phyto.39.1.285 11701867

[CIT0020] Hulse, S. V., Antonovics, J., Hood, M. E., & Bruns, E. L. (2023). Specific resistance prevents the evolution of general resistance and facilitates disease emergence. Journal of Evolutionary Biology, 36(5), 753–763. 10.1111/jeb.14170 36971466 PMC10316961

[CIT0021] Jones, K. E., Patel, N. G., Levy, M. A., Storeygard, A., Balk, D., Gittleman, J. L., & Daszak, P. (2008). Global trends in emerging infectious diseases. Nature, 451(7181), 990–993. 10.1038/nature06536 18288193 PMC5960580

[CIT0022] Korves, T., & Bergelson, J. (2004). A novel cost of r gene resistance in the presence of disease. The American Naturalist, 163(4), 489–504. 10.1086/382552 15122498

[CIT0023] Koskella, B., Lin, D. M., Buckling, A., & Thompson, J. N. (2011). The costs of evolving resistance in heterogeneous parasite environments. Proceedings of the Royal Society B: Biological Sciences, 279(1735), 1896–1903. 10.1098/rspb.2011.2259 PMC3311890 22171085

[CIT0024] Koskella, B., & Parr, N. (2015). The evolution of bacterial resistance against bacteriophages in the horse chestnut phyllosphere is general across both space and time. Philosophical Transactions of the Royal Society of London, Series B: Biological Sciences, 370(1675), 20140297. 10.1098/rstb.2014.0297 26150663 PMC4528495

[CIT0025] Laine, A. -L. (2004). Resistance variation within and among host populations in a plant–pathogen metapopulation: Implications for regional pathogen dynamics. Journal of Ecology, 92(6), 990–1000. 10.1111/j.0022-0477.2004.00925.x

[CIT0026] Laine, A. -L. (2005). Spatial scale of local adaptation in a plant-pathogen metapopulation. Journal of Evolutionary Biology, 18(4), 930–938. 10.1111/j.1420-9101.2005.00933.x 16033565

[CIT0027] Lee, H. -A., Lee, H. -Y., Seo, E., Lee, J., Kim, S. -B., Oh, S., Choi, E., Choi, E., Lee, S. E., & Choi, D. (2017). Current understandings of plant Nonhost resistance. Molecular Plant–Microbe Interactions, 30(1), 5–15. 10.1094/MPMI-10-16-0213-CR 27925500

[CIT0028] Leonard, K. J. (1977). Selection pressures and plant pathogens. Annals of the New York Academy of Sciences, 287(1), 207–222. 10.1111/j.1749-6632.1977.tb34240.x

[CIT0029] Leonard, K. J. (1994). Stability of equilibria in a gene-for-gene coevolution model of host-parasite interactions. Phytopathology (USA), 84(1), 70–77. 10.1094/Phyto-84-70

[CIT0030] Lerner, N., Luizzi, V., Antonovics, J., Bruns, E., & Hood, M. E. (2021). Resistance correlations influence infection by foreign pathogens. The American Naturalist, 198(2), 206–218. 10.1086/715013 PMC8283004 34260867

[CIT0031] Lewis, J. A., Penley, M. J., Sylla, H., Ahumada, S. D., & Morran, L. T. (2022). Antagonistic coevolution limits the range of host defense in *C. elegans* populations. Frontiers in Cellular and Infection Microbiology, 12, 758745.

[CIT0032] Longdon, B., Brockhurst, M. A., Russell, C. A., Welch, J. J., & Jiggins, F. M. (2014). The evolution and genetics of virus host shifts. PLoS Pathogens, 10(11), e1004395. 10.1371/journal.ppat.1004395 25375777 PMC4223060

[CIT0033] Märkle, H., Saur, I. M. L., & Stam, R. (2022). Evolution of resistance (R) gene specificity. Essays in Biochemistry, 66(5), 551–560. 10.1042/EBC20210077 35612398

[CIT0034] Marmor, M., Hertzmark, K., Thomas, S. M., Halkitis, P. N., & Vogler, M. (2006). Resistance to HIV Infection. Journal of Urban Health, 83(1), 5–17. 10.1007/s11524-005-9003-8 16736351 PMC1539443

[CIT0035] McCarthy, A. J., Shaw, M. -A., Jepson, P. D., Brasseur, S. M. J. M., Reijnders, P. J. H., & Goodman, S. J. (2011). Variation in European harbour seal immune response genes and susceptibility to phocine distemper virus (PDV). Infection, Genetics and Evolution, 11(7), 1616–1623. 10.1016/j.meegid.2011.06.002 21712101

[CIT0036] Meyers, B. C., Kaushik, S., & Nandety, R. S. (2005). Evolving disease resistance genes. Current Opinion in Plant Biology, 8(2), 129–134. 10.1016/j.pbi.2005.01.002 15752991

[CIT0037] Miller, M. E., Nazareno, E. S., Rottschaefer, S. M., Riddle, J., Pereira, D. D. S., Li, F., Nguyen-Phuc, H., Henningsen, E. C., Persoons, A., Saunders, D. G. O., Stukenbrock, E., Dodds, P. N., Kianian, S. F., & Figueroa, M. (2020). Increased virulence of *Puccinia coronata* f Spavenae populations through allele frequency changes at multiple putative Avr loci. PLoS Genetics, 16(12), e1009291. 10.1371/journal.pgen.1009291 33370783 PMC7793281

[CIT0038] Mundt, C. C. (2014). Durable resistance: A key to sustainable management of pathogens and pests. Infection, Genetics and Evolution, 27, 446–455. 10.1016/j.meegid.2014.01.011 PMC4117828 24486735

[CIT0039] Nürnberger, T., Brunner, F., Kemmerling, B., & Piater, L. (2004). Innate immunity in plants and animals: Striking similarities and obvious differences. Immunological Reviews, 198(1), 249–266. 10.1111/j.0105-2896.2004.0119.x 15199967

[CIT0040] Parker, B. J., Hrček, J., McLean, A. H. C., & Godfray, H. C. J. (2017). Genotype specificity among hosts, pathogens, and beneficial microbes influences the strength of symbiont-mediated protection. Evolution, 71(5), 1222–1231. 10.1111/evo.13216 28252804 PMC5516205

[CIT0041] Parker, I. M., Saunders, M., Bontrager, M., Weitz, A. P., Hendricks, R., Magarey, R., Suiter, K., & Gilbert, G. S. (2015). Phylogenetic structure and host abundance drive disease pressure in communities. Nature, 520(7548), 542–544. 10.1038/nature14372 25903634

[CIT0042] Poland, J. A., Balint-Kurti, P. J., Wisser, R. J., Pratt, R. C., & Nelson, R. J. (2009). Shades of gray: The world of quantitative disease resistance. Trends in Plant Science, 14(1), 21–29. 10.1016/j.tplants.2008.10.006 19062327

[CIT0043] Powell, M. J. D. (1964). An efficient method for finding the minimum of a function of several variables without calculating derivatives. The Computer Journal, 7(2), 155–162. 10.1093/comjnl/7.2.155

[CIT0044] Ross, R. (1916). An application of the theory of probabilities to the study of a priori pathometry—Part I. Proceedings of the Royal Society of London, Series A: Containing Papers of a Mathematical and Physical Character, 92(638), 204–230. 10.1098/rspa.1916.0007

[CIT0045] Sasaki, A. (2000). Host-parasite coevolution in a multilocus gene-for-gene system. Proceedings of the Royal Society B: Biological Sciences, 267(1458), 2183–2188. 10.1098/rspb.2000.1267 PMC1690804 11413631

[CIT0046] Savage, A. E., & Zamudio, K. R. (2011). MHC genotypes associate with resistance to a frog-killing fungus. Proceedings of the National Academy of Sciences of the United States of America, 108(40), 16705–16710. 10.1073/pnas.1106893108 21949385 PMC3189034

[CIT0047] Streicker, D. G., Turmelle, A. S., Vonhof, M. J., Kuzmin, I. V., McCracken, G. F., & Rupprecht, C. E. (2010). Host phylogeny constrains cross-species emergence and establishment of rabies virus in bats. Science, 329(5992), 676–679. 10.1126/science.1188836 20689015

[CIT0048] Tellier, A., & Brown, J. K. M. (2007). Polymorphism in multilocus host–parasite coevolutionary interactions. Genetics, 177(3), 1777–1790. 10.1534/genetics.107.074393 17947440 PMC2147965

[CIT0049] Thompson, J. N., & Burdon, J. J. (1992). Gene-for-gene coevolution between plants and parasites. Nature, 360(6400), 121–125. 10.1038/360121a0

[CIT0050] Thrall, P. H., Biere, A., & Uyenoyama, M. K. (1995). Frequency-dependent disease transmission and the dynamics of the *Silene*–*Ustilago* host–pathogen system. The American Naturalist, 145(1), 43–62. 10.1086/285727

[CIT0051] Thrall, P. H., & Burdon, J. J. (2003). Evolution of virulence in a plant host-pathogen metapopulation. Science, 299(5613), 1735–1737. 10.1126/science.1080070 12637745

[CIT0052] Thrall, P. H., Burdon, J. J., & Young, A. (2001). Variation in resistance and virulence among demes of a plant host–pathogen metapopulation. Journal of Ecology, 89(5), 736–748. 10.1046/j.0022-0477.2001.00597.x

[CIT0053] Thrall, P. H., Laine, A. -L., Ravensdale, M., Nemri, A., Dodds, P. N., Barrett, L. G., & Burdon, J. J. (2012). Rapid genetic change underpins antagonistic coevolution in a natural host-pathogen metapopulation. Ecology Letters, 15(5), 425–435. 10.1111/j.1461-0248.2012.01749.x 22372578 PMC3319837

[CIT0054] Virtanen, P., Gommers, R., Oliphant, T. E., Haberland, M., Reddy, T., Cournapeau, D., Burovski, E., Peterson, P., Weckesser, W., Bright, J., van der Walt, S. J., Brett, M., Wilson, J., Millman, K. J., Mayorov, N., Nelson, A. R. J., Jones, E., Kern, R., Larson, E., & van Mulbregt, P. (2020). SciPy 10: Fundamental algorithms for scientific computing in Python. Nature Methods, 17(3), Article 3. 10.1038/s41592-019-0686-2 PMC7056641 32094914

